# An Anti-Inflammatory Signature Across Pain and Cognition: Not All Mediterranean Diets Are Equal

**DOI:** 10.3390/nu18121983

**Published:** 2026-06-18

**Authors:** Pablo Maya, Teresa López de Coca, María Aracely Calatayud-Pascual, Elena Grau-García, Roxana González, Fernando Cardona, José Andrés Román, Daniel Ramón, Jordi Pérez-Tur, Lucrecia Moreno

**Affiliations:** 1 Cátedra DeCo MICOF-CEU UCH, Universidad Cardenal Herrera-CEU, CEU Universities, 46115 Valencia, Spain; pablo.maya@alumnos.uchceu.es (P.M.); teresa.lopezperez@uchceu.es (T.L.d.C.); maria.calatayud@uchceu.es (M.A.C.-P.); 2 Department of Pharmacy, Universidad Cardenal Herrera-CEU, CEU Universities, 46115 Valencia, Spain; 3 Rheumatology Department, La Fe University and Polytechnic Hospital, 46026 Valencia, Spain; elena_grau@iislafe.es (E.G.-G.); roman_jan@gva.es (J.A.R.); 4 Rheumatology Research Group, La Fe Health Research Institute, 46026 Valencia, Spain; 5 Rheumatology Service, Valencia University General Hospital, 46014 Valencia, Spain; gonzalez_rox@gva.es; 6 Institut de Biomedicina de València-CSIC, Centro de Investigación Biomédica en Red en Enfermedades Neurodegenerativas (ISCIII), 46010 Valencia, Spain; fcardona@ibv.csic.es; 7 Departamento de Biotecnología, Universitat Politècnica de València, 46022 Valencia, Spain; 8 Medicine Department, Faculty of Medicine, Universitat de València, 46010 Valencia, Spain; 9 Department of Animal Production and Health, Veterinary Public Health, and Food Science and Technology, Universidad Cardenal Herrera-CEU, CEU Universities, 46115 Valencia, Spain; daniel.ramonvidal@uchceu.es

**Keywords:** anti-inflammatory diet, chronic pain, dietary inflammatory index, semantic verbal fluency, subjective memory complaints

## Abstract

**Background**: Chronic pain and early cognitive vulnerability frequently co-occur in older women and may share inflammatory mechanisms. **Objective**: We examined whether dietary inflammatory load, assessed using the dietary inflammatory index (DII), and Mediterranean-derived dietary patterns (Mediterranean diet (MED), Dietary Approaches to Stop Hypertension (DASH), Mediterranean–DASH Intervention for Neurodegenerative Delay (MIND) and anti-inflammatory Mediterranean Diet (AnMED)) are associated with pain and early cognitive outcomes. **Methods**: We conducted a cross-sectional study among women aged ≥50 years recruited from community pharmacies and healthcare centers in the Comunidad Valenciana (Spain). Dietary intake was assessed using the PREDIMED Food Frequency Questionnaire to derive DII and dietary pattern scores. Outcomes included pain intensity, subjective memory complaints (SMC) and semantic verbal fluency (SVF). Analyses were adjusted for sociodemographic, clinical and lifestyle covariates, with false discovery rate correction. **Results**: Complete case samples comprised 470 women for SMC and SVF, with 328 also included for pain. Higher DII was consistently associated with greater pain intensity, increased odds of SMC, and lower SVF scores. No dietary pattern was associated with pain after correction. AnMED was associated with lower odds of SMC and higher SVF, while DASH was also positively associated with SVF. Bridge analysis showed that lower DII was associated with both MIND and AnMED, with a stronger association for AnMED. **Conclusions**: Dietary inflammatory load showed the most consistent associations with pain and early cognitive vulnerability, whereas Mediterranean-derived patterns differed in their inflammatory and cognitive relevance.

## 1. Introduction

Alzheimer’s disease is increasingly conceptualized as a long biological continuum, rather than a disorder with an abrupt clinical onset, in which neuropathological processes may evolve for decades before objective cognitive impairment becomes clinically apparent [1]. During this preclinical window, functional independence is frequently preserved, yet subtle cognitive inefficiencies may already coexist with early biological dysregulation, preceding mild cognitive impairment [1,2]. In this context, subjective memory complaints have gained relevance as an early functional marker of increased cognitive vulnerability and higher progression risk across the Alzheimer’s disease continuum [2,3]. Early cognitive vulnerability is not a unitary construct, and some cognitive domains may be particularly sensitive to lifestyle-related exposures.

This domain-specific framing is highly pertinent in rheumatology and chronic pain medicine. Cognitive complaints often emerge in individuals with chronic inflammatory conditions, where persistent pain coexists with fatigue, sleep, and mood symptoms. Chronic pain and cognitive vulnerability share convergent neurobiological mechanisms— chronic low-grade inflammation, oxidative stress, altered neurotransmitter signaling, central sensitization, and gut microbiota dysbiosis—each implicated in neuroimmune activation and neuroinflammatory processes relevant to cognitive dysfunction and neurodegeneration [4,5,6].

In light of these developments, nutrition has therefore emerged as a leading modifiable factor with the potential to influence both symptom burden and long-term brain health trajectories. Dietary patterns modulate systemic inflammation [7], oxidative stress [8], vascular dysfunction, and neuroinflammation [9], all of which are implicated in both chronic pain and cognitive decline.

Mediterranean-derived dietary patterns have been associated with reduced risk of cognitive decline and dementia [10,11,12,13] and may also be relevant to pain [14]. However, these patterns are not equivalent, as they differ in restriction stringency, component composition, weighting and inclusion of pro-inflammatory foods. The traditional Mediterranean diet (MED) is characterized by high intake of plant-based foods, legumes, nuts, and fish, with extra-virgin olive oil as the main fat source and moderate intakes of dairy, meat, and alcohol [10,11]. The DASH diet prioritizes cardiometabolic health through reduced sodium and saturated fat intake [12]. The MIND diet integrates elements of MED and DASH, prioritizing green leafy vegetables and berries, while maintaining olive oil as the principal fat source [13,14]. By contrast, the AnMED represents a deliberately more restrictive model that classifies foods according to inflammatory potential, penalizing pro-inflammatory foods and emphasizing anti-inflammatory foods [15].

Mechanistically, the gut microbiota is a key interface through which diet may influence immune-metabolic signaling, systemic inflammation, and gut–brain communication [16]. These pathways may affect processes related to both neurodegeneration and chronic pain, including oxidative stress, neuroinflammation, impaired neurotransmission, and barrier dysfunction [17]. Accordingly, anti-inflammatory dietary strategies may provide dual benefits by reducing inflammatory burden while supporting diet-sensitive cognitive functions [18].

Taken together, these observations support a clinically and biologically grounded hypothesis: Mediterranean-derived dietary patterns are not interchangeable, and the magnitude of their association with cognitive vulnerability in chronic pain settings may depend on their capacity to reduce dietary inflammatory potential, an attribute most explicitly targeted by AnMED.

Despite growing interest, several gaps persist that are of particular relevance to rheumatology. First, cognitive vulnerability in chronic pain populations is often addressed descriptively, without dissecting whether specific, clinically meaningful domains show stronger associations with modifiable exposures such as diet. Second, Mediterranean-derived patterns are frequently treated as interchangeable, even though their inflammatory stringency differs substantially. Third, it remains unclear whether a global inflammatory exposure tool, such as the DII or specific Mediterranean-derived pattern scores, better captures the shared signal underlying pain intensity and early cognitive vulnerability. In this context, we examined the associations between DII and four Mediterranean-derived dietary patterns with pain intensity, SMC and SVF in women aged 50 years and older. We focused exclusively on women as chronic pain is more prevalent and disabling in women, making this population particularly relevant for examining the pain–cognition interplay [19]. We hypothesized that DII would show consistent associations with pain and cognition and that AnMED, as the most inflammation-restrictive pattern, would align most strongly with lower DII and more favorable cognitive outcomes.

## 2. Materials and Methods

### 2.1. Study Design and Participants

We carried out a five-month cross-sectional study among women aged 50 years or older recruited through community pharmacies and healthcare centers in the Comunidad Valenciana, Spain. Exclusion criteria were male sex, a diagnosis of dementia or intellectual disability, and severe sensory or physical impairments precluding interview participation.

Sample size considerations were based on the ability to detect regression-based associations with DII as the main exposure, rather than differences between predefined groups. Under two-sided α = 0.05 and 80% power, the required sample size was approximately 151 for NRS-11 and 187 for SMC in unadjusted models. Assuming conservative covariate correlation (R^2^ = 0.40), the size increased to approximately 252 and 312, respectively. SVF was analyzed using negative binomial regression because of its count distribution and overdispersion. As no robust prior assumptions were available for SVF under the planned negative binomial model, the minimum target sample was defined according to the largest formally estimable requirement. Therefore, a minimum of 312 participants was considered adequate to support inference for the prespecified continuous and binary primary outcomes, while SVF was analyzed exploratorily within the same analytical framework.

### 2.2. Data Collection and Clinical Measures

Participants completed face-to-face interviews lasting approximately one hour. Anthropometric measurements were obtained to calculate body mass index (BMI). Participants reported the presence of arterial hypertension, diabetes mellitus, and hyperlipidemia.

Pain intensity was assessed using the 11-point Numeric Rating Scale (NRS-11) [20]. SMC were captured using a dichotomous self-report item (yes/no) on perceived memory complaints, a widely used low-burden indicator of early self-perceived cognitive vulnerability [2,3]. Cognitive performance was assessed using the SVF test [21] since previous studies showed its sensitivity to dietary factors [22]. Depressive symptoms were evaluated using the Geriatric Depression Scale-5 (GDS-5) [23].

With this scheme, we collected SVF and SMC information from 470 individuals whereas NRS-11 was available for 328 women. Missing data was due to the unavailability of those women to perform the scale.

### 2.3. Dietary Assessment and Derivation of Dietary Scores

Dietary intake over the previous month was assessed using the validated PREDIMED Food Frequency Questionnaire (FFQ) [24]. Nutritional information and bioactive content were obtained from the Souci–Fachmann–Kraut Food Composition and Nutrition Tables (9th edition) [25].

FFQ data were used to compute the DII based on the methodology described by Shivappa et al. [26]. Nutrient intakes were standardized, converted to centered percentiles, weighted by published inflammatory response scores, and summed to produce a global DII score, with higher values indicating a more pro-inflammatory dietary profile.

Adherence scores were calculated for four Mediterranean-derived patterns: MED [27], DASH [28], MIND [29], and AnMED [15]. For comparability across patterns, all scores were z-standardized prior to analysis. High adherence was defined using established cut-offs for each pattern [15]. Appendix A Table A1 shows the similarities and differences between the four Mediterranean dietary patterns.

### 2.4. Statistical Analysis

A complete case analysis was performed separately for each outcome. NRS-11 was modeled using linear regression with heteroskedasticity-robust standard errors, SMC using logistic regression, and SVF using negative binomial regression. All dietary pattern scores were z-standardized before modeling. All models were adjusted for age, education, GDS-5, arterial hypertension, diabetes mellitus, hyperlipidemia, BMI, sleep hours, and total energy intake. False discovery rate (FDR) correction was applied using the Benjamini–Hochberg procedure, and statistical inference was primarily based on q-values < 0.05. To address whether apparent differences between Mediterranean-derived dietary patterns were supported by direct statistical evidence, exploratory post hoc pairwise comparisons of pattern-specific effect estimates were performed for each outcome using bootstrap resampling. All analysis were done using R 4.4.2.

## 3. Results

### 3.1. Characteristics of the Population

We have designed a cross-sectional study to observe the effect of diet on pain and cognitive status. Due to its nature, we cannot discard the fact that what we observe could reflect a reverse causation, that is, that the clinical outcomes prompt the patients to follow a specific diet.

A total of 470 women were included in the complete case analyses for SVF and SMC, whereas pain models included 328 women due to missingness in NRS-11. The NRS-11 analytical sample, therefore, represents a complete case subsample of the broader SMC/SVF sample rather than a separate population. The flow diagram of participants is described in Figure S1. Baseline characteristics of the study population and of the outcome-specific analytical samples are shown in Table 1.

Exploratory pairwise comparisons between Mediterranean-derived dietary pattern estimates are shown in Supplementary Table S1. This analysis did not support the global statistical superiority of AnMED or any other Mediterranean-derived pattern across all outcomes. For NRS-11, no pairwise comparison remained significant after FDR correction, although the nominal MIND-DASH comparison reached *p* = 0.024, q = 0.144. For SMC, AnMED showed more favorable estimates than MED and DASH in the expected direction, but these differences did not remain significant after FDR correction (MED-AnMED: difference in log[OR] = 0.266, 95% CI 0.011 to 0.552, *p* = 0.044, q = 0.120; AnMED-DASH: difference in log[OR] = −0.265, 95% CI −0.494 to −0.048, *p* = 0.024, q = 0.120). For SVF, AnMED and DASH showed significantly stronger associations than MIND after FDR correction (AnMED-MIND: difference in log[IRR] = 0.062, 95% CI 0.028 to 0.097, *p* < 0.001, q < 0.001; MIND-DASH: difference in log[IRR] = −0.061, 95% CI −0.103 to −0.021, *p* = 0.002, q = 0.006), whereas AnMED did not differ from DASH (AnMED-DASH: difference in log[IRR] = 0.000, 95% CI −0.038 to 0.038, *p* = 0.980, q = 0.980). Therefore, the observed pattern-specific findings should be interpreted as differences in consistency and direction of associations within this cohort, rather than as definitive evidence that one Mediterranean-derived pattern is globally superior to the others.

### 3.2. Mediterranean Diet (MED)

**Figure 1:**
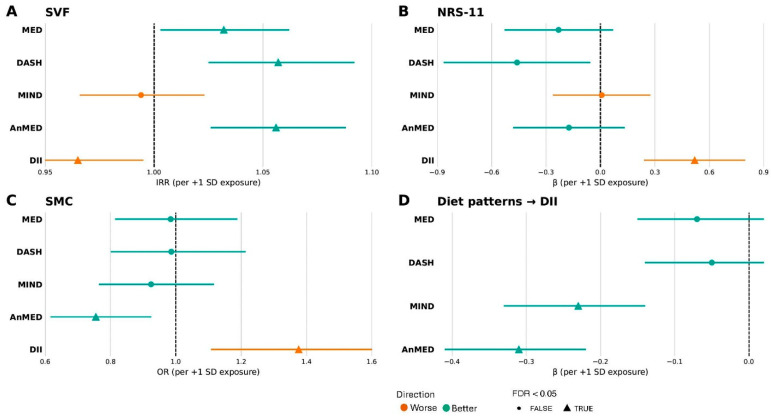
Associations of the dietary inflammatory index and Mediterranean-derived dietary patterns with pain and cognitive outcomes. (**A**) Multivariable-adjusted associations with pain intensity assessed by NRS-11, expressed as β coefficients and 95% confidence intervals. (**B**) Multivariable-adjusted associations with SMC, expressed as odds ratios (OR) and 95% confidence intervals. (**C**) Multivariable-adjusted associations with SVF, expressed as incidence rate ratios (IRR) and 95% confidence intervals. (**D**) Bridge analysis: Associations between Mediterranean-derived dietary patterns and DII. Points show multivariable-adjusted β coefficients and 95% confidence intervals for DII per +1 SD increase in dietary pattern z-scores. Negative β values indicate alignment with a more anti-inflammatory dietary profile. Exposures include the DII and z-standardized Mediterranean-derived dietary pattern scores (MED, DASH, MIND, and AnMED). For Mediterranean-derived dietary patterns, all effect estimates are therefore standardized and represent the expected change per +1 SD increase in adherence score, allowing comparison of effect direction and magnitude across patterns. FDR was controlled using the Benjamini–Hochberg procedure, and triangle markers denote associations with FDR-adjusted q-values < 0.05.

Higher MED adherence showed a modest positive association with SVF (IRR 1.032, 95% CI 1.003 to 1.062; q = 0.044) (Table 2; Figure 1A). No significant associations were observed with NRS-11 (β −0.231, 95% CI −0.529 to 0.068; q = 0.263; Figure 1B) or with SMC (OR 0.984, 95% CI 0.814 to 1.188; q = 0.893; Figure 1C). Thus, MED showed a modest association restricted to verbal fluency.

### 3.3. Dietary Approaches to Stop Hypertension (DASH)

Higher DASH adherence was associated with better SVF performance (IRR 1.057, 95% CI 1.025 to 1.092; q = 0.001) (Table 2; Figure 1A). The nominal association with NRS-11 pain (β −0.460, 95% CI −0.863 to −0.057; *p* = 0.026; Figure 1B) did not remain significant after FDR correction (q = 0.104), and no association was observed with SMC (OR 0.986, 95% CI 0.801 to 1.214; q = 0.893; Figure 1C). Thus, DASH showed a clear positive signal for verbal fluency only.

### 3.4. Mediterranean–DASH Intervention for Neurodegenerative Delay (MIND)

After FDR correction, the MIND pattern showed no significant associations with SVF (IRR 0.994, 95% CI 0.966 to 1.023; q = 0.681), NRS-11 (β 0.007, 95% CI −0.261 to 0.275; q = 0.958), or SMC (OR 0.924, 95% CI 0.765 to 1.116; q = 0.827) (Table 2; Figure 1A–C). Therefore, despite its conceptual orientation toward neuroprotection, MIND did not show detectable associations with the outcomes evaluated in this cohort.

### 3.5. Anti-Inflammatory Mediterranean Diet (AnMED) and Bridge Analysis

Higher adherence to AnMED was associated with higher SVF (IRR 1.056, 95% CI 1.026 to 1.088; q = 0.001) and with lower odds of SMC (OR 0.755, 95% CI 0.617 to 0.925; q = 0.026) (Table 2). No significant association with NRS-11 was observed after FDR correction (β −0.174, 95% CI −0.481 to 0.133; q = 0.356) (Figure 1A–C). Among the Mediterranean-derived dietary patterns examined, AnMED was the only one associated with both lower subjective cognitive vulnerability and better cognitive performance.

To further examine whether this pattern captured the inflammatory dimension of diet, a bridge analysis was performed using DII as the dependent variable. Higher adherence to AnMED was strongly associated with lower DII (β −0.312, 95% CI −0.407 to −0.216; q < 0.001), indicating a more anti-inflammatory dietary profile. MIND was also associated with lower DII (β −0.237, 95% CI −0.335 to −0.139; q < 0.001), whereas MED and DASH were not (Table 3; Figure 1D). Because all dietary scores were derived from the same FFQ, this analysis should be interpreted as an assessment of operational alignment with dietary inflammatory potential, rather than as evidence of an independent biological mechanism. These findings indicate that Mediterranean-derived dietary patterns were not interchangeable in their alignment with dietary inflammatory potential, with AnMED showing the largest inverse association with DII within this construct-alignment analysis.

### 3.6. Food-Group Energy Analysis

**Figure 2:**
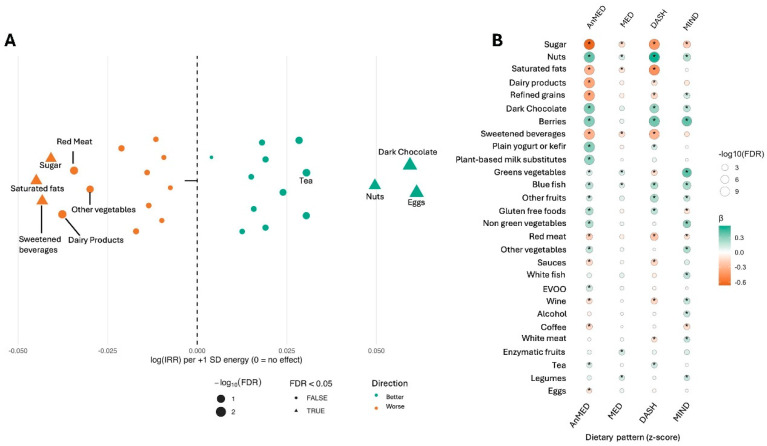
Food-group energy intake associated with SVF and its alignment with Mediterranean-derived dietary patterns. (**A**) Points show log (IRR) for SVF per +1 SD increase in food-group energy intake after log1p transformation and z-score standardization; the dashed vertical line indicates the null effect. Point size represents −log10(FDR), triangles indicate FDR < 0.05, and color denotes direction of association with SVF (better vs. worse). (**B**) Bubble plot showing the association between Mediterranean-derived dietary pattern z-scores and food-group energy intake. Bubble size represents −log10(FDR) with * showing those with FDR < 0.05, and color indicates the direction and magnitude of the association (β).

As a secondary exploratory analysis intended to contextualize the pattern-level findings, food-group energy analysis did not identify any specific food-group associations with NRS-11 or SMC after FDR correction. By contrast, it identified a specific subgroup signature associated with SVF after FDR correction (Table S2; Figure 2A). Higher energy intake from dark chocolate (>85% cocoa) (IRR 1.061, 95% CI 1.031 to 1.092; q = 0.002), eggs (IRR 1.063, 95% CI 1.031 to 1.097; q = 0.002), and nuts (IRR 1.051, 95% CI 1.018 to 1.084; q = 0.017) was associated with higher SVF. In contrast, higher energy intake from saturated fats (IRR 0.956, 95% CI 0.927 to 0.986; q = 0.026), sweetened beverages (IRR 0.958, 95% CI 0.929 to 0.987; q = 0.027), and sugary products (IRR 0.960, 95% CI 0.931 to 0.990; q = 0.043) was associated with lower SVF.

When these SVF-related food groups were examined in relation to dietary patterns, AnMED and DASH showed a coordinated profile characterized by higher energy from SVF-favorable subgroups, particularly nuts and dark chocolate, and lower energy from SVF-unfavorable subgroups, particularly sugary products, sweetened beverages, and saturated fats (Figure 2B). This pattern-level coupling supports a structured dietary signature underlying the verbal fluency findings. and links the food-group analysis to the broader Mediterranean-derived dietary framework, rather than presenting these foods as isolated drivers of the main results.

## 4. Discussion

This study provides a coherent dietary-inflammatory signal linking pain intensity and early cognitive vulnerability in women aged ≥50 years, a population highly relevant to rheumatology practice, where chronic symptoms and cognitive complaints often coexist. The main finding was that DII emerged as the most consistent dietary correlate across all outcomes, linking a more pro-inflammatory diet to higher pain intensity, higher odds of SMC, and lower SVF. Within the Mediterranean-derived patterns, SVF appeared to be the most diet-sensitive cognitive measure, and AnMED showed an operational alignment with lower dietary inflammatory potential and favorable cognitive outcomes in this cohort, without establishing clinical or biological superiority over the other Mediterranean-derived patterns.

A plausible mechanistic interpretation is that dietary inflammatory burden may act upstream of both pain amplification and early cognitive vulnerability through partially overlapping immune-metabolic pathways. A more pro-inflammatory dietary profile has been linked to systemic low-grade inflammation, oxidative stress, vascular dysfunction, neuroinflammatory signaling, and gut–brain immune communication [7,8,9,16,17,18,30,31,32]. These pathways may contribute to pain through peripheral and central sensitization, cytokine-mediated neuronal excitability, and altered nociceptive processing, while also affecting cognition through blood–brain barrier dysfunction, glial activation, synaptic impairment, and vascular–metabolic effects on frontotemporal and executive language networks. Therefore, although the present study cannot demonstrate biological mediation, the observed associations are consistent with a shared inflammatory framework linking diet, pain intensity, and early cognitive vulnerability.

A key point for interpretation is why DII showed robust associations with pain, whereas Mediterranean-derived patterns did not survive FDR correction for this outcome. This discrepancy is more likely methodological than biological in the present dataset. DII captures the inflammatory potential of the whole diet as a continuous cumulative exposure across the dietary matrix [26,32,33], making it better suited to detect dose–response relationships with graded outcomes such as pain intensity under the present FFQ-based operationalization. In contrast, the pattern indices used here are threshold-based adherence scores. They are intentionally designed for interpretability and classification, but they are inherently capped: once a cut-off is reached, additional intake does not translate into a higher score, and individuals with meaningfully different intakes can be assigned the same adherence value. This compression of exposure variability (ceiling effects and discretization) attenuates associations with continuous outcomes like NRS-11, particularly when between-participant differences are driven by quantity and balance rather than the binary achievement of a criterion. This issue becomes even more relevant after multiple testing corrections, especially given the small proportion of participants with high AnMED or DASH adherence. In this context, DII appears to be the most informative metric for pain-related inference, whereas dietary patterns are better interpreted as structured behavioral summaries rather than direct quantitative measures of inflammatory load. However, this interpretation is specific to the scoring algorithms, FFQ instrument, sample distribution, and cross-sectional design used in this cohort, and should not be generalized to all Mediterranean-derived dietary scores or populations without external validation.

Among the Mediterranean-derived patterns, AnMED was associated with both lower odds of SMC and higher SVF and also showed the largest inverse association with DII in the bridge analysis. This is coherent with its design, as AnMED explicitly penalizes pro-inflammatory foods while prioritizing anti-inflammatory components. More broadly, our findings reinforce that Mediterranean-derived patterns are not interchangeable from an operational perspective. However, the present analyses do not provide formal statistical evidence of superiority of one dietary pattern over another. We also acknowledge that the observed effect sizes are modest and often close to the null, which is expected for dietary exposures in multifactorial outcomes such as pain and cognition; therefore, Figure 1 should not be interpreted as evidence that AnMED is substantially different from the other patterns on visual grounds alone. In Mediterranean settings such as ours, where adherence to core Mediterranean elements is relatively common, traditional Mediterranean-based scores may have limited discriminatory capacity. Under these circumstances, operationalizations that incorporate dose–response gradients, capturing the balance between pro- and anti-inflammatory food intake, may better reflect dietary variation relevant to inflammatory symptoms and cognitive vulnerability [10,11,12,13,14]. This measurement perspective also helps explain why the AnMED-DII link is particularly valuable. The bridge analysis suggests, at the operational level, that AnMED is not merely “another Mediterranean score” but the pattern that most consistently maps onto a lower inflammatory dietary profile in this cohort. However, because DII and dietary pattern scores were derived from the same FFQ, this analysis should be viewed as a construct-alignment analysis rather than as direct biological validation or evidence of independent clinical superiority. Notably, this association is observed despite the relatively small number of participants classified as having high adherence to AnMED in this cohort, although this low frequency also warrants cautious interpretation. This has clinical relevance because it suggests that the anti-inflammatory quality of a “Mediterranean” diet depends on how the pattern is operationalized, not simply on the label itself.

Similarly, MIND adherence was associated with lower DII but did not associate with SVF or SMC, suggesting that lowering inflammatory potential, while necessary, may not be sufficient to generate detectable cognitive signals. This may reflect reduced variability in this population rather than the absence of biological relevance, especially given the broader literature supporting cognitive benefits of MIND adherence [13,14,34,35,36]. MED showed a weaker and more selective signal, being associated only with SVF but not with pain or SMC. By contrast, DASH showed a clear association with SVF but not with lower DII, which may reflect its stronger emphasis on vascular and cardiometabolic pathways [12,36,37] rather than on explicit restriction of pro-inflammatory foods.

From the cognitive perspective, SVF may have been the most diet-sensitive cognitive outcome in this cohort, reflecting frontotemporal and executive language efficiency and showing sensitivity to lifestyle exposures [21,22]. Chronic inflammatory conditions frequently show cognitive deficits affecting lexical retrieval and executive control. In this framework, the inverse association between DII and SVF fits with evidence linking cognitive impairment with pro-inflammatory diets [30,38,39], implying that diet is an actionable domain that could modify the path to disease in early cognitive vulnerability.

At the food-subgroup level, dark chocolate, nuts, and eggs, foods rich in polyphenols, unsaturated fats, choline, and neurotrophic nutrients, were positively associated with SVF after FDR correction, supporting a diet-responsive signal in frontotemporal language and executive networks rather than a generic cognitive effect. These analyses should be interpreted as exploratory and complementary to the dietary-pattern results, helping to identify which food-group components may contribute to the SVF-related profile observed for AnMED and DASH. In the case of dark chocolate, cocoa flavanols and polyphenols have been linked to vascular and neuroprotective mechanisms (e.g., improved cerebral perfusion, reduced oxidative stress, and modulation of neurotrophic signaling), and controlled interventions with cocoa products have reported benefits in cognition-relevant outcomes [38,39]. As for nuts, the evidence supports the association with cognitive trajectories and domain-level performance, including endpoints related to verbal fluency [40,41]. In the case of eggs, the signal is mechanistically consistent with their content of choline (acetylcholine synthesis), carotenoids, and essential fatty acids, and longitudinal and observational studies link egg consumption with improved cognitive performance [42,43]. However, this external evidence should be considered mechanistic and epidemiological context rather than direct confirmation of the present food-subgroup findings, because study populations, exposure definitions, doses, product composition, and follow-up periods differ from the present cross-sectional FFQ-based setting together, these findings suggest a coherent SVF-favorable dietary signature plausibly linked to antioxidant, anti-inflammatory, and vascular-neurotrophic pathways, consistent with previous works [44,45,46,47,48,49].

However, the food-level interpretation should explicitly avoid a simplistic good vs. bad foods narrative. A plausible explanation, consistent with dietary behavior in older adults, is that some subgroups associated with poorer SVF may operate as markers of dietary displacement and reduced dietary diversity. Higher energy from sugary products, sweetened beverages, and saturated-fat-rich items often indicates a diet where energy allocation crowds out vegetables, legumes, and micronutrient-dense foods, resulting in lower variability and a narrower repertoire of protective components. In that scenario, the observed association is not only attributable to adverse biological effects of specific foods but also to the absence of protective dietary elements that would otherwise support cognitive performance. This displacement framework fits well with the pattern-mapping results, where patterns associated with higher SVF (AnMED and DASH) show coordinated alignment with SVF-favorable subgroups and inverse alignment with SVF-unfavorable subgroups, reinforcing that the signal is pattern-consistent rather than driven by isolated food items. Overall, these findings support a clinically interpretable whole-diet signature rather than single-food attribution.

The pain findings further strengthen the clinical relevance of these results as the DII tracked pain intensity, whereas pattern scores did not remain significant after FDR correction. This is consistent with broader literature showing associations between pro-inflammatory dietary profiles and musculoskeletal pain phenotypes and with evidence in chronic pain linking pro-inflammatory diet to higher pain and disease severity [29]. The present results add granularity by showing that the same inflammatory dietary dimension is also linked to early cognitive vulnerability, a comorbidity that is clinically meaningful in chronic symptom management and may affect daily function and quality of life [30]. These findings support anti-inflammatory dietary counseling as a low-risk, scalable adjunctive strategy in chronic inflammatory conditions, not as a substitute for medical therapy, but as a modifiable upstream exposure aligned with shared inflammatory biology.

The cross-sectional design precludes causal inference and is susceptible to reverse causation. Specifically, women with greater pain intensity, functional limitation, fatigue, or cognitive complaints may have modified their dietary choices or had a reduced capacity to maintain structured dietary patterns. Therefore, the observed associations should be interpreted as non-causal and hypothesis-generating. Dietary assessment relied on FFQ data—information sensitive to recall bias. Thus, misreporting or inaccurate recall of habitual intake may have affected the estimation of food-group consumption, total energy intake, and derived dietary scores. As previously noted, pattern indices are threshold-based and therefore less sensitive to dose–response effects on continuous outcomes; limited variability in high-adherence categories also reduces power for pattern-level pain inference, particularly for AnMED and DASH patterns, with few participants classified as high-adherent. This imbalance may also reduce the precision and stability of regression estimates, especially for AnMED, and limit the extent to which findings can be generalized to individuals with sustained high adherence to this restrictive anti-inflammatory pattern. Although models were adjusted for key covariates, residual confounding is possible. Objective inflammatory biomarkers were not available. Strengths of the study include face-to-face data collection, comprehensive dietary characterization, and concurrent assessment of pain and cognitive outcomes. Finally, although the female-only sample is clinically justified by the higher burden of chronic pain in women, this epidemiological rationale does not necessarily imply that the diet–cognition associations observed here are female-specific or directly transferable to cognitive outcomes in men. Future studies should include men to determine whether these associations are sex-specific or generalizable across sexes.

## 5. Conclusions

In summary, this study suggests that dietary inflammatory load may represent a shared dietary-inflammatory signal associated with pain intensity and early cognitive vulnerability in older women. DII emerged as the most informative exposure metric for pain-related inference, while AnMED was the pattern that showed operational alignment with favorable cognitive outcomes and lower DII in this cohort. In rheumatology and chronic pain settings, our findings provide an interpretable framework and suggest that dietary inflammatory load may represent a relevant and modifiable target in older women. An explicitly anti-inflammatory Mediterranean patterning may therefore warrant further evaluation as a structured nutritional approach across the pain–cognition interface, without implying global superiority over other Mediterranean-derived patterns, as direct pairwise comparisons did not support such an interpretation across all outcomes.

## Figures and Tables

**Table 1:** Baseline characteristics of the study population and outcome-specific analytical samples (SMC/SVF and NRS-11).

Variables		SMC and SVF N = 470	NRS-11 N = 328
		Mean ± SD/n (%)	Mean ± SD/n (%)
Age (years)		68.67 ± 11.90	65.77 ± 10.83
Education	Illiterate	1 (0.22%)	1 (0.31%)
	Read and write	23 (4.89%)	19 (5.79%)
	Primary education	71 (15.11%)	53 (16.16%)
	Secondary education	156 (33.19%)	110 (33.54%)
	Higher education	219 (46.59%)	145 (44.21%)
GDS-5	No	420 (89.37%)	281 (85.68%)
	Yes	50 (10.63%)	47 (14.32%)
AHT	No	288 (61.3%)	214 (65.24%)
	Yes	182 (38.72%)	114 (34.76%)
DM	No	413 (87.87%)	289 (88.11%)
	Yes	57 (12.13%)	39 (11.89%)
HLP	No	312 (66.38%)	218 (66.46%)
	Yes	158 (33.62%)	110 (33.54%)
RA	No	437 (92.98%)	293 (89.33%)
	Yes	33 (7.02%)	35 (10.67%)
HC	No	421 (89.57%)	279 (85.06%)
	Yes	49 (10.53%)	49 (14.94%)
Sleep hours		6.59 ± 1.35	6.52 ± 1.44
BMI (kg/m^2^)		26.94 ± 4.86	27.07 ± 4.76
SMC	No	199 (42.34%)	150 (45.73%)
	Yes	271 (57.66%)	178 (54.27%)
SVF		18.70 ± 7.05	20.20 ± 7.33
NRS-11		3.48 ± 2.95	3.53 ± 2.95
MED	Low adherence (<9)	162 (34.49%)	125 (38.11%)
	High adherence (≥9)	308 (65.53%)	203 (61.89%)
DASH	Low adherence (<7.5)	412 (87.66%)	279 (85.06%)
	High adherence (≥7.5)	58 (12.34%)	49 (14.94%)
MIND	Low adherence (<9)	67 (14.26%)	57 (17.38%)
	High adherence (≥9)	403 (85.75%)	271 (82.62%)
AnMED	Low adherence (<117)	453 (96.38%)	311 (94.82%)
	High adherence (≥117)	17 (3.62%)	17 (5.18%)
Total energy (kcal)		1837.99 ± 763.74	1968.60 ± 851.55
Total fat (g)		106.46 ± 50.88	114.50 ± 52.99
Total protein (g)		58.32 ± 25.19	61.01 ± 29.68
Total carbohydrates (g)		134.57 ± 85.99	144.92 ± 100.39
Total fiber (g)		30.04 ± 16.11	32.66 ± 18.50

The pain sample is a complete case subsample of the larger SMC/SVF analytical sample, restricted by missingness in pain data. Therefore, data are presented as mean ± standard deviation for continuous variables and as counts (n) and frequencies (%) for categorical variables. Two outcome-specific complete case samples are shown: the sample used for analyses involving SMC and SVF (n = 470), and the sample used for analyses involving pain intensity assessed with NRS-11 (n = 328). AHT, arterial hypertension; DM, diabetes mellitus; HLP, hyperlipidemia; GDS-5, Geriatric Depression Scale; BMI, body mass index; RA, rheumatoid arthritis; HC, healthy controls. Total energy, fat, protein, carbohydrates, and fiber represent the mean daily dietary intake.

**Table 2:** Association between dietary inflammatory index and dietary patterns with clinical and cognitive outcomes.

Exposure	SVF, n = 470		NRS-11, n = 328		SMC, n = 470	
	IRR, (95% CI)		β, (95% CI)		OR, (95% CI)	
	*p*	q	*p*	q	*p*	q
MED	1.032 (1.003; 1.062)		−0.231 (−0.529; 0.068)		0.984 (0.814;1.188)	
	0.033	**0.044**	0.131	0.263	0.866	0.893
DASH	1.057 (1.025; 1.092)		−0.460 (−0.863; 0.057)		0.986 (0.801; 1.214)	
	<0.001	**<0.001**	0.026	0.104	0.893	0.893
MIND	0.994 (0.966; 1.023)		0.007 (−0.261; 0.275)		0.924 (0.765; 1.116)	
	0.681	0.681	0.958	0.958	0.413	0.827
AnMED	1.056 (1.034; 1.088)		−0.174 (−0.481; 0.133)		0.755 (0.617; 0.925)	
	<0.001	**<0.001**	0.267	0.356	0.007	**0.026**
DII	0.965 (0.936; 0.995)		0.519 (0.241; 0.797)		1.376 (1.108; 1.708)	
	0.021	**0.021**	<0.001	**<0.001**	0.003	**0.004**

Exposures are reported on their modeling scale: DII per 1-unit increase; Mediterranean-derived pattern scores (MED, DASH, MIND, AnMED) per 1 SD increase (z-scores). Score distributions in this cohort (Mean ± SD): MED-14 9.137 ± 1.807; DASH 4.872 ± 1.666; MIND 10.128 ± 1.388; AnMED 41.376 ± 33.738; DII −0.785 ± 1.299. Primary inference: q < 0.05. Significant associations are shown in bold. Abbreviations: OR, odds ratio; IRR, incidence rate ratio; NRS-11, Numeric Rating Scale; CI, confidence interval; SMC, subjective memory complaints; SVF, semantic verbal fluency.

**Table 3:** Dietary patterns and their association with DII.

Exposure n = 470	β (95% CI)	*p*	q
MED	−0.070 (−0.166; 0.027)	0.158	0.210
DASH	−0.053 (−0.146; 0.041)	0.270	0.270
MIND	−0.237 (−0.335; −0.139)	<0.001	**<0.001**
AnMED	−0.312 (−0.407; −0.216)	<0.001	**<0.001**

Multivariable-adjusted associations of Mediterranean-derived dietary pattern z-scores with DII (β per +1 SD; 95% CI). Primary inference: q < 0.05. Significant associations after FDR adjustment are shown in bold.

## Data Availability

Data described in the manuscript, code book, and analytic code will be made available upon reasonable request.

## Supplementary Materials

The following supporting information can be downloaded at: https://www.mdpi.com/article/10.3390/nu18121983/s1, Table S1. Exploratory pairwise comparisons of Mediterranean-derived dietary pattern effect estimates; Table S2. Multivariable-adjusted associations between food-group energy intake and NRS-11, SMC, and SVF; Figure S1. Participant flow diagram.

## Abbreviations

The following abbreviations are used in this manuscript:

AHT	Arterial hypertension
AnMED	Anti-inflammatory Mediterranean Diet
BMI	Body mass index
DASH	Dietary Approaches to Stop Hypertension
DII	Dietary inflammatory index
DMT	Diabetes mellitus
EVOO	Extra-virgin olive oil
FDR	False discovery rate
FFQ	PREDIMED Food Frequency Questionnaire
GDS-5	Geriatric depression scale, version 5
HC	Healthy controls
HLP	Hyperlipidemia
MED	Mediterranean pattern
MIND	Mediterranean–DASH Intervention for Neurodegenerative Delay
NRS-11	11-point Numeric Rating Scale
RA	Rheumatoid arthritis
SMC	Subjective memory complaints
SVF	Semantic verbal fluency

## Appendix A

**Table A1:** Conceptual and operational differences across Mediterranean-derived dietary patterns.

	MED	DASH	MIND	AnMED
Alcohol: Wine	7 spw ^1^	≤1 spd	7 spw ^1^	Not allowed
Beverages: Coffee	No guidelines	No guidelines	No guidelines	≤2 spd
Beverages: Sweetened or carbonated	<1 spd	<5 spw	<1 spd	Not allowed
Dairy products	No guidelines	≥2 spd ^2^	<1 spd ^3^	Restricted ^4^
Fats: EVOO	≥4 tspd	No guidelines ^5^	Primary oil use ^6^	3 tspd
Fats: Saturated	<7 spw	<6% tei	<7 spw	Not allowed
Fish	≥3 spw	<2 spd	≥1 spw	≥3 spw ^7^
Fruits	≥3 spd	≥4 spd	≥2 spw ^8^	1 daily piece ^9^
Grains	No guidelines	Allowed ^10^	No guidelines	Not allowed
Legumes	≥3 spw	≥4 spw	≥3 spw	≥3 spw
Meat: White	No guidelines ^11^	<2 spd	≥2 spw.	≥3 spw
Meat: Red	≤1 spd	<2 spw	<4 spw.	Not allowed
Nuts	≥3 spd	≥4 spw	≥5 spw	1 spd
Sugar and pastries	<2 spw	<5 spw	<5 spw	Not allowed
Turmeric	No guidelines	No guidelines	No guidelines	5 g/day
Vegetables	≥2 spd	≥4 spd	Green leafy: ≥6 spw Other: daily.	100 g daily ^12^

Green, foods recommended within the dietary pattern; yellow, foods allowed in limited or moderate amounts; red, foods whose consumption is not recommended. EVOO: extra-virgin olive oil; tei: total energy intake; tspd: tablespoon per day (15 mL); spd: servings per day; spw: servings per week. All reference amounts are reported in [24]. ^1^ Glasses or red wine. ^2^ Preferably, low-fat. ^3^ Daily ration of milk. Occasional cheese, not weekly. ^4^ Cow milk not allowed. Cured cheese or goat/sheep cheese, natural yogurt or kefir preferred. ^5^ Priority given to unsaturated fats. ^6^ No daily recommendations. ^7^ Servings per week of each white and blue fish. ^8^ Refer to berries exclusively. ^9^ Enzymatic, antioxidant and other fruits: one piece of each group daily. ^10^ Priority for whole grains. ^11^ Preferential consumption of red meat. ^12^ Green, non-green or other vegetables. Amount of each of the three groups.
