# Supplementary material for: An Anti-Inflammatory Signature Across Pain and Cognition: Not All Mediterranean Diets Are Equal
Source: Nutrients. 2026 Jun 18;18(12):1983. doi: 10.3390/nu18121983 (PMC13306077; doi:10.3390/nu18121983)
Supplement: Supplementary file 1 [file nutrients-18-01983-s001.zip › Supplementary Materials Maya-Lopez de Coca.pdf]

Supplementary Materials.

Supplementary Table S1. Exploratory pairwise comparisons of Mediterranean-derived dietary pattern effect estimates.

Outcome	Contrast	Difference	(95% CI)	p	q
**NRS-11**	MED-AnMED	-0.054	(-0.462; 0.296)	0.724	0.724
	MED-MIND	-0.234	(-0.598; 0.107)	0.160	0.320
	MED-DASH	0.233	(-0.229; 0.674)	0.332	0.398
	AnMED-MIND	-0.181	(-0.477; 0.132)	0.226	0.339
	AnMED-DASH	0.287	(-0.121; 0.733)	0.160	0.320
	MIND-DASH	0.467	(0.048; 0.924)	0.024	0.144
**SMC**	MED-AnMED	0.266	(0.011; 0.552)	0.044	0.120
	MED-MIND	0.065	(-0.155; 0.300)	0.570	0.734
	MED-DASH	0.001	(-0.250; 0.264)	0.998	0.998
	AnMED-MIND	-0.201	(-0.445; 0.010)	0.060	0.120
	AnMED-DASH	-0.265	(-0.494; -0.048)	0.024	0.120
	MIND-DASH	-0.064	(-0.322; 0.197)	0.612	0.734
**SVF**	MED-AnMED	-0.022	(-0.061; 0.013)	0.228	0.342
	MED-MIND	0.039	(0.004; 0.074)	0.026	0.052
	MED-DASH	-0.022	(-0.062; 0.016)	0.300	0.360
	AnMED-MIND	0.062	(0.028; 0.097)	<0.001	**<0.001**
	AnMED-DASH	0.000	(-0.038; 0.038)	0.980	0.980
	MIND-DASH	-0.061	(-0.103; -0.021)	0.002	**0.006**

Pairwise comparisons were performed using bootstrap resampling. For NRS-11, values represent differences in β coefficients. For SMC, values represent differences in log(OR). For SVF, values represent differences in log(IRR). q-values correspond to Benjamini-Hochberg FDR-adjusted p-values. In bold, significant results.

Supplementary Table S2. Multivariable-adjusted associations between food-group energy intake and NRS-11, SMC, and SVF.

Exposure	**Pain, n=328** β, (95% CI)		**SMC, n=470** OR, (95% CI)		**SVF, n=470** IRR, (95% CI)	
	**p**	**q**	**p**	**q**	**p**	**q**
**Alcohol: Other**	0.001 (-0.326; 0.328)		0.932 (0.774-1.124)		1.024 (0.995; 1.054)	
	0.996	0.996	0.462	0.719	0.101	0.218
**Alcohol: Wine**	0.011 (-0.310; 0.332)		1.047 (0.864; 1.270)		1.019 (0.990; 1.049)	
	0.947	0.996	0.636	0.810	0.199	0.349
**Beverages: Coffee**	0.356 (0.044; 0.669)		1.111 (0.916; 1.348)		1.016 (0.986; 1.046)	
	0.026	0.184	0.284	0.638	0.295	0.435
**Beverages: Sweetened or carbonated**	0.282 (-0.054; 0.617)		0.989 (0.812; 1.205)		0.958 (0.929; 0.987)	
	0.101	0.354	0.915	0.965	0.005	**0.027**
**Beverages: Tea**	-0.246 (-0.560; 0.068)		1.022 (0.844; 1.239)		1.031 (1.002; 1.061)	
	0.126	0.392	0.821	0.919	0.037	0.116
**Dairy products**	0.004 (-0.334; 0.341)		1.280 (1.048; 1.564)		0.963 (0.935; 0.992)	
	0.983	0.996	0.016	0.279	0.014	0.056
**Dark chocolate**	-0.175 (-0.509; 0.159)		0.908 (0.748; 1.102)		1.061 (1.031; 1.092)	
	0.306	0.543	0.327	0.638	<0.001	**0.002**
**Eggs**	-0.483 (-0.844; -0.123)		1.065 (0.867; 1.308)		1.063 (1.031; 1.097)	
	0.009	0.135	0.551	0.772	<0.001	**0.002**
**Fats: EVOO**	0.219 (-0.156; 0.594)		0.902 (0.728; 1.118)		1.031 (0.997; 1.066)	
	0.254	0.543	0.347	0.638	0.072	0.169
**Fats: Saturated**	0.297 (-0.031; 0.625)		1.238 (1.009; 1.519)		0.956 (0.927; 0.986)	
	0.077	0.354	0.041	0.384	0.004	**0.026**
**Fish: Blue**	-0.076 (-0.414; 0.261)		0.906 (0.746; 1.101)		1.013 (0.983; 1.043)	
	0.657	0.856	0.323	0.638	0.406	0.494
**Fish: White**	0.069 (-0.278; 0.417)		1.180 (0.967; 1.438)		1.004 (0.974; 1.034)	
	0.695	0.856	0.103	0.439	0.795	0.795
**Fruits: Berries**	-0.095 (-0.445; 0.255)		0.976 (0.801; 1.190)		1.018 (0.988; 1.050)	
	0.597	0.856	0.812	0.919	0.242	0.376
**Fruits: Enzymatic**	-0.247 (-0.607; 0.113)		0.973 (0.792; 1.195)		0.990 (0.960; 1.021)	
	0.179	0.502	0.793	0.919	0.531	0.595
**Fruits: Other**	-0.355 (-0.704; -0.005)		0.910 (0.742; 1.116)		0.987 (0.957; 1.017)	
	0.048	0.266	0.364	0.638	0.382	0.486
**Gluten-free foods**	0.189 (-0.154; 0.531)		0.933 (0.764; 1.139)		1.015 (0.985; 1.046)	
	0.281	0.543	0.495	0.730	0.328	0.460
**Grains: Refined**	-0.432 (-0.758; -0.105)		1.184 (0.973; 1.442)		0.979 (0.950; 1.009)	
	0.010	0.135	0.092	0.439	0.165	0.331
**Legumes**	-0.133 (-0.454; 0.188)		0.861 (0.706; 1.050)		0.983 (0.956; 1.011)	

	0.418	0.688	0.140	0.490	0.239	0.376
**Meat: Red**	-0.168 (-0.481; 0.144)		0.991 (0.816; 1.204)		0.966 (0.938; 0.995)	
	0.291	0.543	0.930	0.965	0.022	0.077
**Meat: White**	0.214 (-0.113; 0.541)		0.896 (0.731; 1.098)		0.989 (0.960; 1.018)	
	0.201	0.812	0.288	0.638	0.443	0.517
**Nuts**	-0.413 (-0.742; -0.084)		0.780 (0.633; 0.962)		1.051 (1.018; 1.084)	
	0.014	0.135	0.020	0.279	0.002	**0.017**
**Plain yogurt**	0.015 (-0.297; 0.327)		0.950 (0.786; 1.148)		1.019 (0.990; 1.049)	
**or kefir**	0.927	0.996	0.594	0.792	0.199	0.349
**Plant-based**	0.122 (-0.209; 0.453)		0.838 (0.689; 1.019)		1.029 (0.999; 1.060)	
**milk substitutes**	0.470	0.731	0.076	0.439	0.058	0.148
**Sauces**	0.162 (-0.151; 0.476)		1.002 (0.824; 1.219)		0.986 (0.957; 1.016)	
	0.310	0.543	0.982	0.982	0.363	0.484
**Sugar**	0.026 (-0.322; 0.375)		1.109 (0.908; 1.356)		0.960 (0.931; 0.990)	
	0.883	0.996	0.310	0.638	0.009	**0.043**
**Vegetables:**	-0.066 (-0.358; 0.226)		0.918 (0.751; 1.121)		0.993 (0.963; 1.023)	
**Green**	0.658	0.856	0.399	0.657	0.624	0.647
**Vegetables:**	-0.306 (-0.664; 0.052)		0.855 (0.686; 1.065)		0.991 (0.960; 1.023)	
**Non-green**	0.095	0.354	0.161	0.502	0.566	0.610
**Vegetables:**	-0.067 (-0.411; 0.277)		0.841 (0.681; 1.040)		0.971 (0.941; 1.001)	
**Other**	0.703	0.856	0.110	0.439	0.055	0.148

Food-group exposures represent energy intake (kcal) aggregated by subgroup from the FFQ and were analyzed after log(1+x) transformation and z-score standardization; effect estimates are reported per 1 SD increase in each exposure. NRS-11 was modelled using robust linear regression (β, 95% CI); SMC was modelled using logistic regression (OR, 95% CI); SVF was modelled using negative binomial regression (IRR, 95% CI). Models were adjusted for age, educational level, depressive symptoms (GDS-5), arterial hypertension (AHT), diabetes mellitus (DM), hyperlipidemia (HLP), BMI, sleep hours, and total daily energy intake. p-values are two-sided; q-values correspond to Benjamini-Hochberg FDR-adjusted p-values.
