# Supplementary figures and images for: An Anti-Inflammatory Signature Across Pain and Cognition: Not All Mediterranean Diets Are Equal
Source: Nutrients. 2026 Jun 18;18(12):1983. doi: 10.3390/nu18121983 (PMC13306077; doi:10.3390/nu18121983)

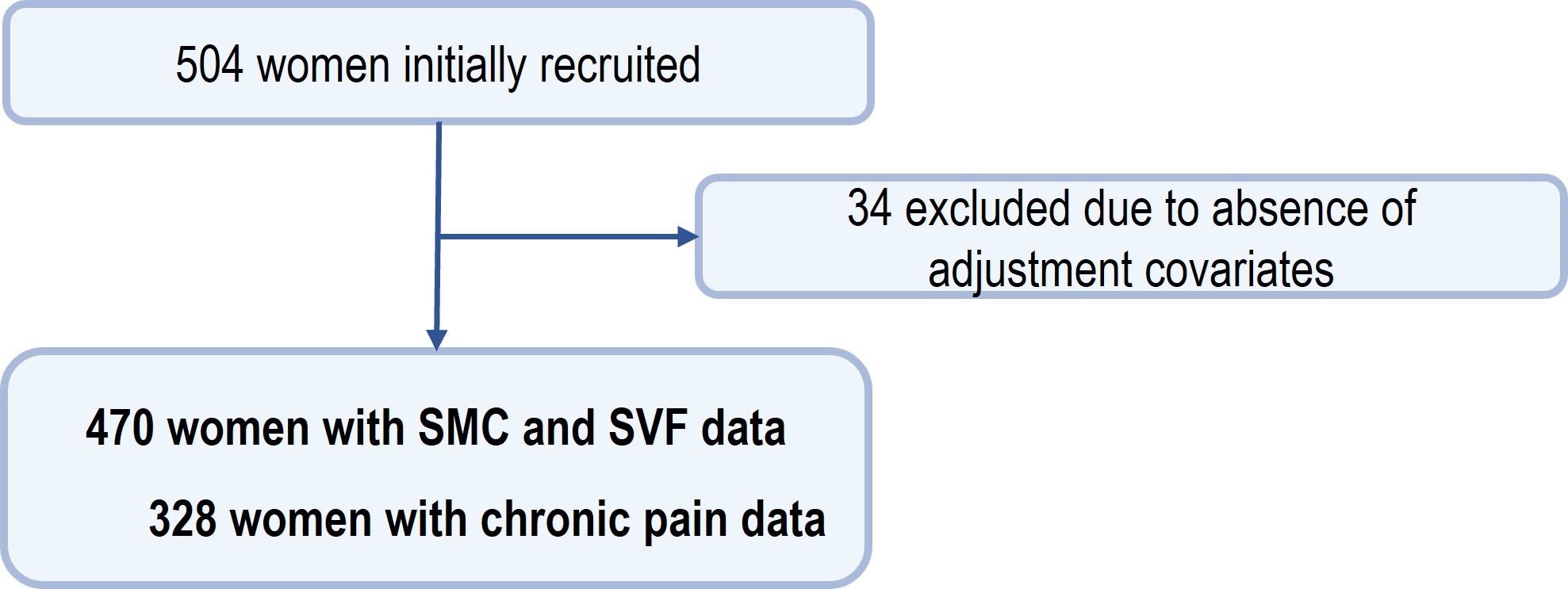

Supplement: Supplementary file 1 [file nutrients-18-01983-s001.zip › Figure S1 Maya-Lopez de Coca.jpg]
